# Developing an HIV Vaccine: The Role of Efficacy Studies in Nonhuman Primates

**DOI:** 10.1371/journal.pmed.0020119

**Published:** 2005-04-26

**Authors:** Klaus Überla

**Affiliations:** **1**Ruhr-University BochumBochumGermany

Given the scientific hurdles encountered in HIV/AIDS vaccine development, a global effort is needed, and the scientific strategic plan proposed by the Global HIV/AIDS Vaccine Enterprise [1] is a milestone. The plan also provides an important starting point for continued discussion.

Since only a limited number of HIV/AIDS vaccines can be tested for efficacy in phase-III studies, evidence-based criteria for selection of candidate HIV/AIDS vaccines for these trials have to be defined. If an immune correlate of protection were available, small-scale immunogenicity studies in humans would provide required parameters. However, the specific immune responses needed for a successful HIV/AIDS vaccine remain unknown. The Global HIV/AIDS Vaccine Enterprise prioritizes research on vaccines eliciting neutralizing antibodies and T cell responses [1]. Standardization of laboratory assays measuring these parameters is proposed to allow comparison of different vaccines.

The main reasons to assume that T cell responses and neutralizing antibodies are important for HIV/AIDS vaccine efficacy are the following: (i) T cell responses and neutralizing antibodies are known to have a role in preventing infection or disease by other viruses, (ii) there is an inverse correlation of T cell responses with viral load in patients with HIV, (iii) depletion of CD8^+^ T cells in nonhuman primate models of AIDS increases viral load, and (iv) passive immunization can provide protection in some of the nonhuman primate models [2]. However, extensive studies during the last 15 years using various T cell assays have failed to provide an accepted immune correlate of protection in nonhuman primates. Although more sophisticated assays and larger groups of animals might reveal such a correlate, protection could also be mediated by a mechanism not yet defined and therefore not monitored.

While the Vaccine Enterprise proposes to search for correlates of immune protection in nonhuman primate models of AIDS, efficacy studies in these models are not mentioned [1]. The limitations of these models have been extensively discussed [3], and there is no proof that these models predict efficacy in humans. However, if one accepts that nonhuman primate models are valid for determination of immune correlates of protection, it seems reasonable to also assume that a more effective vaccine approach in an appropriate nonhuman primate model would also be more effective in humans. Thus, by comparing efficacies of different vaccines in these models, it should be possible to select the most promising vaccine approaches for clinical evaluation. In the past, the nonhuman primate models have been little informative with respect to relative vaccine efficacy: the results from different laboratories were difficult to compare because of differences in, among other things, the monkey species, the inoculation route and dose, the pathogenicity of the challenge virus, the homology between vaccine and challenge virus, and the read-out assays used. This problem was recognized almost ten years ago already, but no agreement has yet been reached on which models most closely resemble HIV transmission and infection in humans. Different aspects of vaccine research require different animal models. In addition, worldwide use of one or two selected models would lead to a shortage in the monkey species needed for these models. Therefore, it is unlikely that an agreement can be reached at all. Rather than going through great and costly efforts to standardize the animal models and laboratory assays, a standardized “state of the art” vaccine approach could be included as a control group in each study. The immunogenicity and efficacy of all novel vaccine candidates could then be determined relative to the immunogenicity and efficacy of the vaccine standard.

A number of issues would need to be discussed with respect to a generally acceptable vaccine standard. Ideally, results from a human efficacy study with the standardized vaccine approach should be available in near future. The standardized vaccine approach should be based on one of the most promising vaccines available at present. Due to the diversity of immunodeficiency viruses used in various nonhuman primate models of AIDS, the vaccine standard cannot be a single vaccine, but must be a standardized vaccine approach. For example, the vaccine approach could be defined by subcutaneous immunization of monkeys with a defined dose of a defined viral vector expressing codon-optimized *gag* and *env* genes at 24 and eight weeks before challenge. The degree of homology of the encoded Gag and Env proteins of the standard vaccine and the challenge virus should be the same as the homology between the antigens of the novel vaccine to be tested and those of the challenge virus. Thus, depending on the novel vaccines to be tested, different vaccine standards of the standardized vaccine approach are needed.

Including defined vaccine standards in future efficacy studies in nonhuman primate models would greatly facilitate preclinical evaluation of novel vaccine candidates and provide evidence-based criteria for their selection for clinical studies. Once an agreement on a standard vaccine approach has been reached, the approach could be implemented quickly, since efficacy studies in nonhuman primates are well established. Given the urgent need for an HIV/AIDS vaccine, we cannot afford to ignore the only animal models that might well predict efficacy in humans. Exploitation of the potential of carefully controlled comparative efficacy studies in nonhuman primates should be considered by the Global HIV/AIDS Vaccine Enterprise. It remains to be discussed whether inclusion of a vaccine standard in clinical studies might also solve some of the standardization problems encountered.
